# Cortical activation of neuromuscular electrical stimulation synchronized mirror neuron rehabilitation strategies: an fNIRS study

**DOI:** 10.3389/fneur.2023.1232436

**Published:** 2023-08-04

**Authors:** Yao Cui, Fang Cong, Fubiao Huang, Ming Zeng, Ruxiu Yan

**Affiliations:** ^1^Department of Physical Therapy, Beijing Bo’ai Hospital, China Rehabilitation Research Center, Beijing, China; ^2^School of Rehabilitation Medicine, Capital Medical University, Beijing, China; ^3^Department of Occupational Therapy, Beijing Bo’ai Hospital, China Rehabilitation Research Center, Beijing, China; ^4^Department of Rehabilitation Medicine, The Second Affiliated Hospital of Jiaxing University, The Second Hospital of Jiaxing City, Jiaxing, Zhejiang, China

**Keywords:** mirror neuron, functional near-infrared spectroscopy, neuromuscular electrical stimulation, action observation, brain-computer interface

## Abstract

**Background:**

The mirror neuron system (MNS) plays a key role in the neural mechanism underlying motor learning and neural plasticity. Action observation (AO), action execution (AE), and a combination of both, known as action imitation (AI), are the most commonly used rehabilitation strategies based on MNS. It is possible to enhance the cortical activation area and amplitude by combining traditional neuromuscular electrical stimulation (NMES) with other top-down and active rehabilitation strategies based on the MNS theory.

**Objective:**

This study aimed to explore the cortical activation patterns induced by NMES synchronized with rehabilitation strategies based on MNS, namely NMES+AO, NMES+AE, and NMES+AI. In addition, the study aimed to assess the feasibility of these three novel rehabilitative treatments in order to provide insights and evidence for the design, implementation, and application of brain-computer interfaces.

**Methods:**

A total of 70 healthy adults were recruited from July 2022 to February 2023, and 66 of them were finally included in the analysis. The cortical activation patterns during NMES+AO, NMES+AE, and NMES+AI were detected using the functional Near-Infrared Spectroscopy (fNIRS) technique. The action to be observed, executed, or imitated was right wrist and hand extension, and two square-shaped NMES electrodes were placed on the right extensor digitorum communis. A block design was adopted to evaluate the activation intensity of the left MNS brain regions.

**Results:**

General linear model results showed that compared with the control condition, the number of channels significantly activated (*P*_FDR_ < 0.05) in the NMES+AO, NMES+AE, and NMES+AI conditions were 3, 9, and 9, respectively. Region of interest (ROI) analysis showed that 2 ROIs were significantly activated (*P*_FDR_ < 0.05) in the NMES+AO condition, including BA6 and BA44; 5 ROIs were significantly activated in the NMES+AE condition, including BA6, BA40, BA44, BA45, and BA46; and 6 ROIs were significantly activated in the NMES+AI condition, including BA6, BA7, BA40, BA44, BA45, and BA46.

**Conclusion:**

The MNS was activated during neuromuscular electrical stimulation combined with an AO, AE, and AI intervention. The synchronous application of NMES and mirror neuron rehabilitation strategies is feasible in clinical rehabilitation. The fNIRS signal patterns observed in this study could be used to develop brain-computer interface and neurofeedback therapy rehabilitation devices.

## 1. Introduction

Upper limb and hand motor dysfunction seriously affect the activities of daily living (ADL) of patients with neurological conditions, such as spinal cord injury (SCI), cerebral palsy (CP), traumatic brain injury (TBI), and stroke survivors, thereby causing severe disability and reducing their quality of life (QOL) (1, 2). The theory of neuroplasticity, which highlights the nervous system’s capacity to alter its structure and function in response to external stimuli, is the basis of neurorehabilitation, and this fundamental concept serves as the basis for various rehabilitative treatments (3). As a common physical therapy method, it has been hypothesized that electrical stimulation (ES) works through a sensorimotor coupling mechanism in which increased proprioceptive signals from evoked movements activate the somatosensory cortex, thereby increasing the excitability of motor cortex neurons, which plays an important role in upper limb and hand functional rehabilitation (4, 5).

Neuromuscular ES (NMES) acts on motor nerves in different body parts by low-frequency current pulses through various surface electrodes (5, 6). The intensity of NMES is set above the motor threshold to induce involuntary movements and facilitate motor function rehabilitation (7, 8). Besides activating muscle fibers, NMES also concurrently activates sensory neurons (8). NMES could have an effect on both muscles and the brain, as it can help to improve the range of motion (ROM) of different joints, strengthen muscles, prevent and improve disuse muscle atrophy, and promote neuroplasticity (5–7). With the development of neuroimaging studies, NMES and functional ES (FES) have been proven as successful methods for stimulating activity-dependent plasticity in brain circuitry, and changes in corticospinal excitability evoked by NMES and FES have been reported (9, 10). Previously published studies have revealed widespread brain activation regions and patterns in response to NMES or FES, including the primary somatosensory cortex (S1), secondary somatosensory area (S2), primary sensory-motor cortex (SM1), primary motor cortex (M1), supplementary motor area (SMA), premotor cortex (PMC), prefrontal cortex (PFC), anterior cingulate cortex (ACC), and cerebellum (3, 4, 10). Brain connections of the above brain regions with other related brain areas and corticospinal projections engaged by NMES have also been reported (6, 9, 11). It has been shown that the effects of sensorimotor activity modulation are related to the intensity of NMES, and that an apparent dose-effect relationship exists between them (6).

Although NMES has many advantages, it is a passive treatment with some disadvantages, including limited active participation, low patient interest in therapy, and easy fatigue (12). It is possible to overcome the above shortcomings and improve the effects by using NMES in synchrony with voluntary exercise (4). According to a previous study, compared to using NMES alone, conducting NMES in synchrony with motor imagery or observation increased the amplitude of motor evoked potentials (MEPs) of upper limb muscles (9). Therefore, it is possible to improve the brain activation area and amplitude by combing NMES with other top-down and active rehabilitation strategies or methods, thus making full use of the rehabilitation time window to achieve better treatment effects, especially for early rehabilitation phases. The mirror neuron system (MNS) plays a key role in the neural mechanism underlying motor learning-related neural plasticity, and a series of rehabilitation strategies and treatment techniques based on MNS theory have been widely applied in patients with neurological conditions and musculoskeletal disease (13, 14). Mirror Neuron (MN) is a special type of neuron that is activated not only when a primate performs a movement, but also when the same or comparable action is seen (15). As a milestone in neuroscience, brain science, cognitive science, and psychology, MNS is crucial in rehabilitation medicine because it provides an “observation-execution matching mechanism” that unifies “action-perception” and “action-execution (AE)” and is important for key neurophysiological processes such as action comprehension, action imitation (AI), motor imagination, and motor learning and relearning (16, 17). The above physiological processes are the important theoretical basis for rehabilitation treatment strategies and methods including action observation therapy (AOT), mirror therapy (MT), motor imagery (MI), brain-computer interface (BCI) rehabilitative robots/ES, and virtual reality (VR) in neural rehabilitation (18).

According to neuroimaging studies, multiple brain regions in the frontal, temporal, and parietal lobes form the human MNS. The MNS network is composed mainly of the posterior inferior frontal gyrus (IFG), ventral premotor cortex (PMC), and rostral inferior parietal lobule (IPL) (19–21). Our previous studies have demonstrated that the clinical application of MNS theory-based rehabilitative methods in stroke patients to improve motor and swallowing function is feasible and effective (17, 22, 23). Among various rehabilitation strategies based on MNS, action observation (AO), AE, and AI are the most commonly used (24). In physical therapy practice, the above MNS-based rehabilitation strategies are usually used in combination. For example, observation and following the performance of target movements are commonly used in therapeutic exercise practice (14). In addition, a published article showed that brain regions activated by NMES and FES overlap with the cortical areas that participate in AE and MI processes (9, 25).

In clinical practice, multiple rehabilitation treatment methods are often used simultaneously to achieve better effects. Based on the above theoretical basis and experimental results, we speculate that the application of NMES with synchronous MNS-based therapies may yield increased and enhanced activation of brain areas and promote the rehabilitation of upper limb and hand motor function, thus achieving synergistic effects. During the NMES treatment, if patients observe (NMES+AO), execute (NMES+AE), or imitate (NMES+AI) the actions produced by the corresponding muscles, it is possible to further promote active participation and MNS activation and promote brain plasticity. Therefore, this study hypothesized that NMES with synchronous MNS-based rehabilitation techniques could result in brain-muscle synchronous intervention. This intervention may enhance the activation of MNS and the recruitment of motor units (MU), thereby improving neuromuscular control function and the rehabilitation effect by promoting neuroplasticity.

To investigate the above hypothesis, we used functional near-infrared spectroscopy (fNIRS) to detect cortical activation patterns linked to these three new treatment techniques and rehabilitation strategies. As a relatively new brain functional imaging method based on the neurovascular coupling theory, fNIRS indirectly reflects neural activity by monitoring the hemodynamic response (HDR) or blood oxygen changes in the human brain cortex through optical technology (26–29). The basic principle of neurovascular coupling theory is that when neurons are activated, oxygen demand increases, subsequently leading to an increase in cerebral blood flow (CBF) (30). Near-infrared (NIR) light in the wavelength range of 700–900 nm exhibits high scattering properties and low absorption rates within human tissue, which allows fNIRS to penetrate deep into brain tissue through hair, scalp, and skull (31). Although fNIRS is commonly used to monitor CBF changes, it can also detect various tissue perfusion patterns using different combinations of source and detector pairs in theoretical and practical applications (32). Human CBF changes induced by specific stimuli/tasks can be measured by fNIRS, and the indexes typically assessed in fNIRS include oxygenated hemoglobin (HbO), deoxygenated hemoglobin (HbR), and total hemoglobin (HbT) (33, 34). CBF increases in the stimulus/task state and decreases in the resting state, and fNIRS is used to evaluate CBF changes by the detection of blood-oxygen-level dependent (BOLD) optical signal changes. Compared with other brain functional imaging techniques, fNIRS has the following advantages: high temporal resolution (~10 Hz), good spatial resolution (2–3 cm), being non-invasive or non-traumatic, real-time monitoring, easy continuous and repeated measurement, low cost, miniaturization, portability, anti-motion artifacts, suitable for a wider range of individuals, and easy to combine with other techniques for multimodal functional imaging, including functional magnetic resonance imaging (fMRI), positron emission tomography (PET), magnetoencephalography (MEG), electroencephalography (EEG), event-related potential (ERP), or electromyography (EMG) (33, 35–37). fNIRS experiments can be carried out in the natural environment without constraints and large amplitude movement monitoring (38). Thus, given its technical properties, especially the anti-motor artifact property, fNIRS has merged as an ideal brain functional imaging techniques for detecting MNS activation. As a result, it is widely used in psychology, pedagogy, rehabilitation medicine, and other fields (18, 39, 40).

The clinical applications of fNIRS in BCI, neural interface (NI), and neurofeedback have increased due to its practical advantages and ability to perform multiple longitudinal acquisitions for monitoring brain activity and neuroplasticity (33, 41). fNIRS neural signal processing or decoding is useful to enhance human-machine interaction and NI for a variety of rehabilitation devices including prosthetics, exoskeleton robotics, and FES, especially for fNIRS-based BCI development in both healthcare and rehabilitation. In theory, EEG, fMRI, MEG, and fNIRS signals can all be used as signal sources for BCI (42). In practice, EEG-BCI has been utilized in rehabilitation scenarios for decades, NIRS has a higher spatial resolution compared to EEG and a lower price and portability compared to fMRI and MEG, but further research is still needed for fNIRS-BCI (43). An fNIRS-BCI based on brain activity induced by MNS rehabilitation strategies combined with NMES-induced haptic feedback is feasible (43). By transforming cortical activity into ES in the hand muscles, BCI helps to connect the neural system and muscles, thereby improving hand rehabilitation (44). MNS-based rehabilitation strategies combined with FES have applied this principle for the design and implementation of BCI, which could promote the activation of the MNS brain region (18). For example, a 2D animation game training system based on video AO and SSVEP triggering BCI-FES rehabilitation action observation for upper limb motor function was designed according to MNS theory. In addition, an experimental study on cerebral cortex excitability of healthy young people was conducted using EEG, which proved that the synergistic effect on cortex excitability in different parts was enhanced under the action of MNS, thereby clarifying the feasibility of such BCI equipment (45).

In summary, this study aimed to use fNIRS to explore the cortical activation patterns induced by NMES synchronized with rehabilitation strategies based on MNS, including NMES+AO, NMES+AE, and NMES+AI. In addition, the study sought to assess the feasibility of these three novel rehabilitative treatments in order to provide insights and evidence for the design, implementation, and application of BCI.

## 2. Materials and methods

### 2.1. Participants

The participants were recruited from China Rehabilitation Research Center, Rehabilitation School of Capital Medical University, and nearby communities. A total of 70 healthy adults were selected from July 2022 to April 2023. After excluding participants with poor fNIRS signal quality or poor task performance, 66 participants were included in the final analysis, and their sociodemographic characteristics are shown in Table 1.

**Table 1:** Demographic and characteristics of the participants.

Characteristic	*N* = 66^1^
Age (years)	23.00 (22.00, 26.00)
**Gender**	
Female	27 (41%)
Male	39 (59%)
Weight (kg)	69.96 ± 14.88
Height (m)	1.71 ± 0.08
BMI (kg/m^2^)	23.75 ± 3.99
**BMI category**	
Normal weight	38 (57.58%)
Obesity	5 (7.58%)
Overweight	17 (25.76%)
Underweight	6 (9.09%)
**Race**	
Han	64 (97%)
Uygur	2 (3%)

^1^n(%); Median (IQR); Mean ± SD. BMI, body mass index. BMI category: Underweight ≤ 18.5 kg/m^2^, normal weight = 18.5–24.9 kg/m^2^, overweight = 25–29.9 kg/m^2^, obesity ≥ 30 kg/m^2^.

The inclusion criteria were as follows: (1) healthy adults (18–60 years old); (2) right-handed, confirmed by Edinburgh Handedness Inventory (EHI); (3) no visual impairment, able to see the videos and text on the screen; (4) no history of neurological or mental illness; (5) education level above junior high school; (6) no history of drug and alcohol abuse; (7) no other diseases that may affect brain structure and function; (8) no serious systemic disease such as heart, lung, liver, and kidney failures. Exclusion criteria: (1) contraindications for NMES intervention: skin injuries or skin diseases (such as eczema), acute infections (such as osteomyelitis), vascular diseases (such as thrombosis or phlebitis), metal implants, and pacemakers; (2) poor fNIRS signal quality caused by thicker hair or unwilling to undergo testing because the fNIRS cap is too tight; (3) fracture, joint injury, muscle pain, and other problems of the right upper limb in the past 3 months; (4) a history of neurological, psychiatric, or musculoskeletal disorders that may affect the execution of experimental tasks; (5) difficulty communicating to complete experimental tasks; (6) taking psychotropic drugs or drinking alcohol within 1 week before the experiment. Those experiencing discomfort or are unwilling to continue undergoing the test can voluntarily withdraw.

This study was approved by the Medical Ethics Committee of the China Rehabilitation Research Center (approval number: 2021-053-1). This study was registered in the Medical Research Registration Information System of the National Health Security Information Platform (Registration number: MR-11-22-013785). All participants signed the informed consent form according to the Helsinki Declaration.

### 2.2. Experimental design and procedure

**Figure 1:**
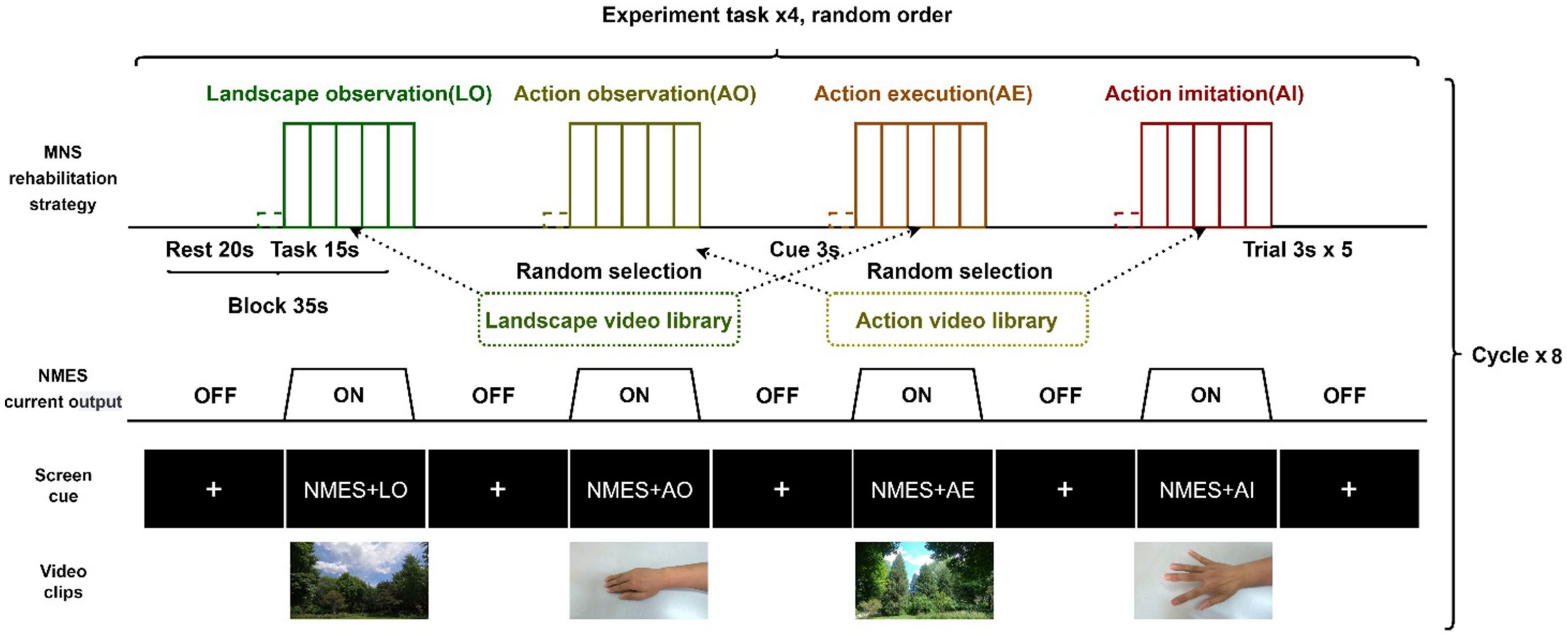
Block experiment design schematic diagram. NMES, neuromuscular electrical stimulation; MNS, mirror neuron system.

A block experimental design with four different tasks was used in this study: NMES+landscape observation (LO), NMES+AO, NMES+AE, and NMES+AI, among which NMES+LO was the control condition. Each block lasted 35 s, including 20 s of rest. During the rest period, the symbol of the fixation point (+) was shown on the screen of the presentation computer, and the participant sat comfortably and relaxed while watching the screen. The task phase of a block lasted 15 s, and during this period, five video clips were randomly selected from the action or landscape video library and played on the presentation computer screen. The participant performed the right wrist and finger extension action or stayed still, or had their right hand passively moved by NMES according to the task cues. We used Python 3.8.10 to write experimental computer programs to accurately control the presentation of videos, instructions, and cues (46). The schematic diagram of the experimental design, screen cue, and video screenshots are shown in Figure 1.

The whole experiment consisted of two stages: preliminary practice and formal test. Before the formal test, the experimenter systematically introduced the experimental content to the participants and prepared them. Before the formal test, the participants were trained using the training program until they fully mastered the test procedure and experimental tasks. The test was conducted in a quiet, dark treatment room with only one experimenter and one participant present. Two digital cameras were used for video recording and the completion of the experimental task was recorded on-site. During the whole experiment, the participant tried to keep still and avoid coughing, swallowing, and sneezing.

In the formal test, the four experimental tasks were cycled eight times. There were eight blocks for each experimental task, totaling 32 blocks, and each block contained five 3-s trials. The rest time at the beginning and end of each test lasted 30 s. In general, a formal test lasted approximately 20 min. To eliminate the influence of the sequence effect, the design principle of randomization was adopted in this study: at the block level, the four experimental tasks in each cycle were randomly ordered; at the trial level, the selection and presentation of action and scenery video clips in a block were done randomly. In the task period, the computer program randomly selected five video clips from the pre-recorded action video library and the landscape video library according to the task type. The action video library had ten right-hand and wrist extension action videos recorded from different angles, and the landscape action library had ten landscape videos. Each video clip lasted 3 s and had a 1080P resolution and 120fps frame rate.

### 2.3. fNIRS data acquisition

#### 2.3.1. fNIRS acquisition

The NIRSport2 fNIRS acquisition device (NIRx Medical Technologies, Minneapolis, United States) with eight sources (S) and eight detectors (D) was used in this experiment. The type of light source of this device was LED, the wavelengths were 769 nm and 850 nm, and the sampling rate was set at 10 Hz. The data recording software was Aurora fNIRS version 2021.9.0.6 (NIRx Medical Technologies, Minneapolis, USA). In the process of fNIRS data collection, the time marker at the beginning of each task was sent to the Aurora fNIRS data acquisition software through WIFI by the Lab Streaming Layer (LSL) signals generated by the PsychoPy program (47).

#### 2.3.2. Montage design

**Figure 2:**
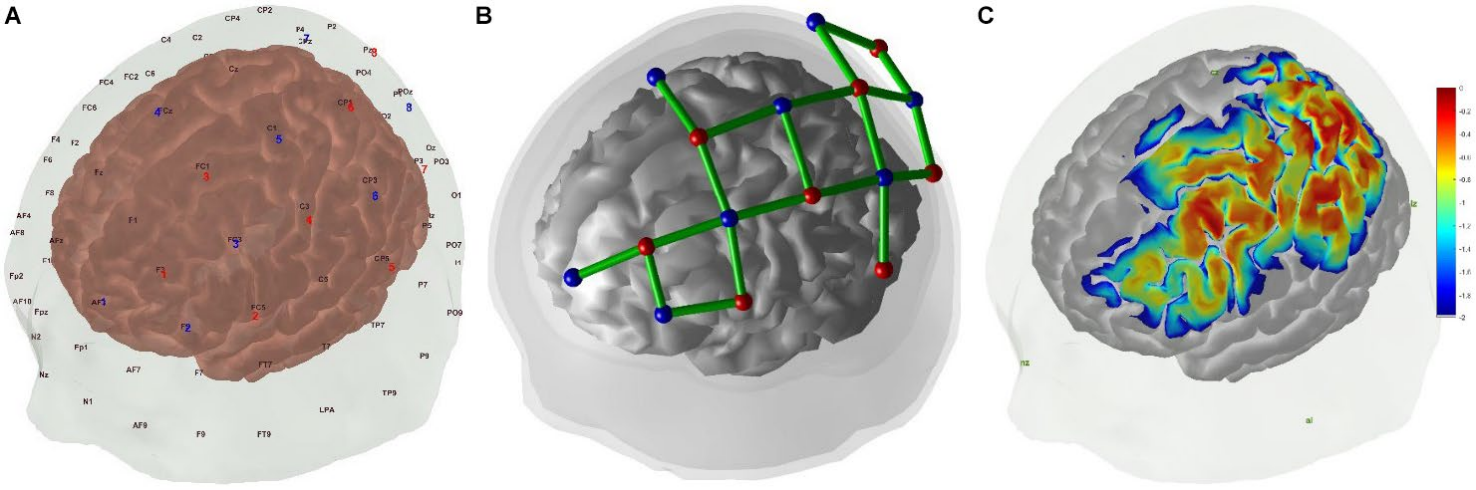
**(A)** Positions of fNIRS optodes over the scalp with the international 10–20 coordinate system, red for sources and blue for detectors. **(B)** fNIRS channels montage in 3D positions over scalp and brain, red for sources, blue for detectors, and green for channels. **(C)** Sensitivity map generated by AtlasViewer package, unit: log_10_(mm^−1^).

The regions of interest (ROIs) in this study were left MNS, including left PMC (BA6), SPL (BA7), IPL (BA40), IFG (BA44/45), and other related brain regions. Based on previous studies, we designed the montage to cover left MNS according to the international 10/20 system (Figure 2A) (48, 49). The montage was designed using the fOLD package and NIRSite software version 2021.4 (NIRx Medical Technologies, Minneapolis, United States) (50). A 20-channel cap montage was designed to cover the ROIs, and we used plastic links to limit the S-D distance of each channel to <3 cm (Figure 2B). Before the experiment, we used the Monte Carlo photon simulation algorithm provided by the AtlasViewer package (51, 52) to simulate the photon path and the measurement sensitivity of the brain regions covered by the montage, thus confirming that ROIs could be detected by the fNIRS brain imaging technique (Figure 2C). Channels were represented in the form of S-D; for example, S1-D1 represented the channel formed by the first source and the first detector. Based on the optodes registered in the Colin-27 atlas, the Montreal Neurological Institute (MNI) coordinates and depth and spatial weights of the ROIs of each channel were calculated and shown in Table 2 (31, 53).

**Table 2:** MNI coordinates, depth, and spatial weights of ROI of the fNIRS channels based on registration to the Colin-27 atlas.

Channel	MNI coordinates			Depth (mm)	Spatial weights of ROI					
	X	Y	Z		BA6	BA7	BA40	BA44	BA45	BA46
S1-D1	−40.028	82.122	29.995	26.501	0.00%	0.00%	0.00%	0.00%	0.00%	9.00%
S1-D2	−55.094	68.108	24.682	20.592	0.00%	0.00%	0.00%	2.19%	49.95%	64.65%
S1-D3	−53.044	50.848	42.793	21.260	0.00%	0.00%	0.00%	17.87%	5.31%	26.36%
S2-D2	−69.006	52.147	12.293	19.889	4.95%	0.00%	0.00%	43.55%	40.34%	0.00%
S2-D3	−67.058	34.678	30.363	18.382	46.58%	0.00%	0.00%	36.39%	4.39%	0.00%
S3-D3	−43.666	32.288	59.292	23.451	7.92%	0.00%	0.00%	0.00%	0.00%	0.00%
S3-D4	−10.188	31.269	75.013	24.716	4.50%	0.00%	0.00%	0.00%	0.00%	0.00%
S3-D5	−30.482	11.346	73.230	20.170	17.89%	0.00%	0.00%	0.00%	0.00%	0.00%
S4-D3	−62.067	14.253	48.546	21.681	13.05%	0.00%	0.00%	0.00%	0.00%	0.00%
S4-D5	−49.037	−6.785	62.588	23.176	5.12%	0.00%	2.89%	0.00%	0.00%	0.00%
S4-D6	−64.562	−23.221	46.414	19.983	0.00%	0.00%	44.54%	0.00%	0.00%	0.00%
S5-D6	−72.278	−37.100	25.118	19.872	0.00%	0.00%	28.81%	0.00%	0.00%	0.00%
S6-D5	−31.636	−28.475	71.383	23.443	0.00%	17.82%	0.66%	0.00%	0.00%	0.00%
S6-D6	−47.704	−45.059	55.802	21.682	0.00%	18.10%	14.22%	0.00%	0.00%	0.00%
S6-D7	−11.015	−49.032	71.726	27.577	0.00%	2.09%	0.00%	0.00%	0.00%	0.00%
S6-D8	−28.389	−63.383	55.839	23.815	0.00%	15.44%	0.00%	0.00%	0.00%	0.00%
S7-D6	−58.204	−56.894	34.052	24.029	0.00%	16.15%	8.89%	0.00%	0.00%	0.00%
S7-D8	−38.971	−75.034	34.184	25.066	0.00%	15.23%	0.00%	0.00%	0.00%	0.00%
S8-D7	8.628	−65.081	61.791	32.014	0.00%	0.00%	0.00%	0.00%	0.00%	0.00%
S8-D8	−8.732	−78.971	45.761	25.181	0.00%	15.19%	0.00%	0.00%	0.00%	0.00%

MNI, Montreal Neurological Institute; BA, Brodmann area; ROI, region of interest; S, Source; D, Detector. The fNIRS montage was registered to the Colin27 atlas, which was used in combination with the automatic anatomical labeling toolbox (aal2) to label the left BA6, BA7, BA40, BA44, BA45, and BA46 (31). Contrast weights were used for the definition of the six ROIs based on the relative sensitivity of each channel to the region derived from the optical forward model (31).

### 2.4. NMES protocol and procedure

In this study, a personal treatment protocol was designed to synchronize the electrical current output with the visual stimuli. The main parameters of the personal protocol were as follows: treatment time: 19 min; waveform: rectangular; phase duration: 200 μs; pulse frequency: 50 Hz; burst frequency: 1 Hz; ramp up time: 1 s; hold time: 15 s; ramp down time: 1 s; interval time: 18 s. We used the EN-STIM 4 (ENRAF-NONIUS, Netherland) NMES device to conduct this study, and one of the four channels was used to conduct the experiment in each session.

In this study, the participant adopted a relaxed and comfortable upright position facing the presentation computer screen. After cleaning the skin and sterilizing it with medical alcohol, we attached two square-shaped unipolar electrodes (Axelgaard Mfg. Co., Ltd., United States) (50 *×* 50 mm) on the right extensor digitorum communis (EDC). The cathode was placed over the motor points of the target muscle, and the anode was placed on the right forearm near the wrist (4). The participant performed the target action or felt movement feeling induced by NMES (43). Before each test, the NMES amplitude was adjusted for each participant to induce finger extension and minimize discomfort, and other parameters were kept constant across all participants. If necessary, the tester carefully placed the electrodes and fixed the cables to prevent electrode detachment. The average amplitude of stimulation for the EDC was 10.55 (9.25, 12.00) mA with a range from 8 to 19 mA. To show the experimental scenario, the photos taken during the experiment of one participant are shown in Supplementary Figure S1.

### 2.5. Data analysis

#### 2.5.1. fNIRS data pre-processing and analysis

In the pre-processing stage, the fNIRS raw data (light intensity) collected by the device was first converted into optical density to present optical absorption. Subsequently, hemoglobin concentration changes were calculated according to the modified Beer–Lambert law (mBLL) (54). Since HbO was a more sensitive index for CBF change, we used the HbO data as markers of cortical activity to build models during the final analysis (3).

In the fNIRS data analysis stage, a general linear model (GLM) was used to detect the relationship between brain activity patterns and the timing of stimulations (31, 55). First, individual-level analysis was carried out, and after pretreatment of original data, autoregressive iteratively reweighted least squares (AR-IWLS) GLM algorithms were used to quantitatively analyze the relationship between HDR and experimental stimulus on each channel of each participant and the regression coefficient (β) and other parameters were obtained for statistical analysis. Then, in the group-level analysis stage, we used a linear mixed-effect model based on the analysis results of the individual-level analysis to determine the relationship between fNIRS brain signals and stimulus timing. The following contrasts were investigated: (1) NMES+AO versus NMES+LO, (2) NMES+AE versus NMES+LO, and (3) NMES+AI versus NMES+LO. The participants were treated as random effects, and the degree of brain activation on each channel was quantitatively analyzed. The estimated value of β and its corresponding standard error were calculated. Subsequently, a *t*-test was used to determine whether β significantly deviated from 0. The false discovery rate (FDR) method was used to obtain multiple comparison correction *p*-values (*P*_FDR_) (56). The average was calculated based on the probability registration method, and the activation results of each ROI were obtained. Finally, the fNIRS optodes were registered on the Colin27 standard brain using the registration algorithm based on the 10–20 system for 3D visualization and analysis. Then, the brain activation maps for channel and ROI analysis compared with the control condition were calculated (53, 57).

All analysis in this section was done in MatLab version R2017b (MathWorks, Massachusetts, United States), and the NIRS Brain AnalyzIR Toolbox was used for fNIRS data pre-processing and statistical modeling (53).

#### 2.5.2. Statistical analysis

For continuous variables, the Shapiro–Wilk normality test was conducted. If it was normally distributed, the variable was described as mean ± standard deviation; otherwise, it was described as median (IQR). Categorical variables were expressed as *n* (%). All the statistical analyses were performed in R version 4.2.3 (58) and RStudio version 023.03.1 (Posit Software, Boston, United States).

## 3. Results

### 3.1. Channels with significant activation compared with the control condition

**Figure 3:**
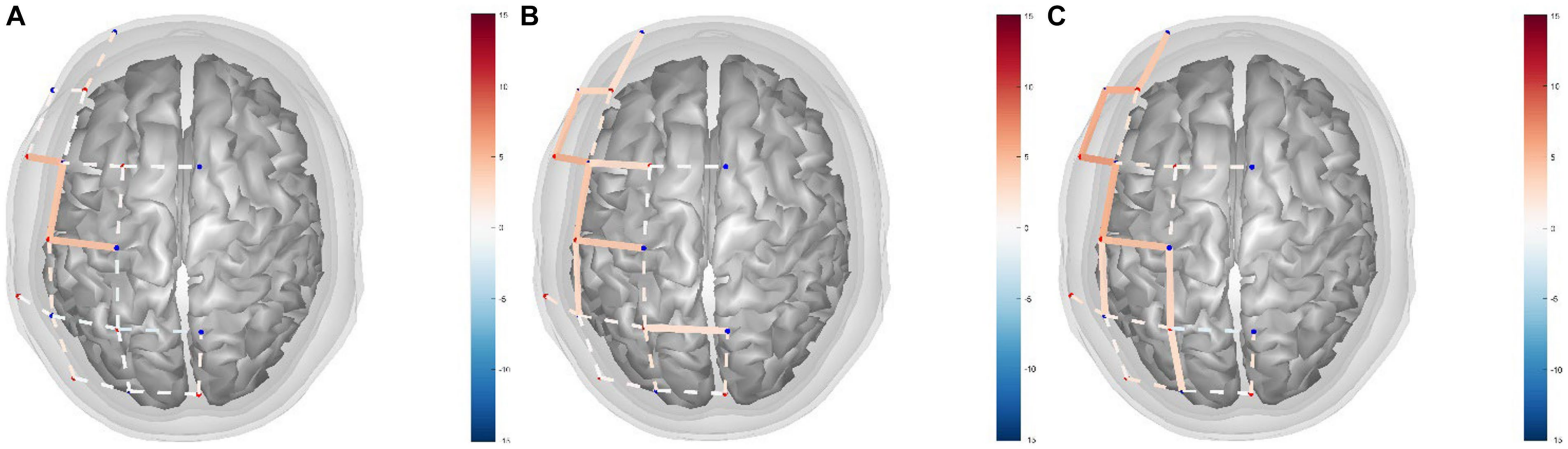
fNIRS channel activity maps based on HbO data for **(A)** NMES+AO versus NMES+LO, **(B)** NMES+AE versus NMES+LO, **(C)** NMES+AI versus NMES+LO. The color of the channel/line indicates the T-statistic according to the color bar (right) with solid lines showing channels significant at a false-discovery rate of *P*_FDR_ < 0.05 corrected for all comparisons (31, 53). FDR, false discovery rate; HbO, oxygenated hemoglobin; NMES, neuromuscular electrical stimulation; AO, action observation; AE, action execution; AI, action imitation; LO, landscape observation.

By performing GLM analysis on the HbO data and using the NMES+LO condition as the control condition, the regression coefficients (β) of each channel in different experimental conditions were obtained (Table 3; Figure 3). The statistical test was conducted and the *t*-value was used for three-dimensional data visualization analysis (Figure 3). The data showed that, compared with the NMES+LO condition, 3 channels were significantly activated (*P*_FDR_ < 0.05) in the NMES+AO condition: S2-D3, S4-D3, and S4-D5 (Figure 3A). In the NMES+AE condition, 9 channels were significantly activated (*P*_FDR_ < 0.05): S1-D1, S1-D2, S2-D2, S2-D3, S3-D3, S4-D3, S4-D5, S4-D6, and S6-D7 (Figure 3B). In the NMES+AI condition, 9 channels were activated (*P*_FDR_ < 0.05): S1-D1, S1-D2, S2-D2, S2-D3, S4-D3, S4-D5, S4-D6, S6-D5, and S6-D8 (Figure 3C). For most channels, the activation amplitude from high to low in turn was NMES+AI, NMES+AE, and NMES+AO (Table 3; Figure 3).

**Table 3:** Channels with significant increases (*P*_FDR_ < 0.05) in HbO concentration compared with the control condition.

Channel	Beta (ꭒM)	SE	*t*-value	*p*-value	*P*_FDR_	Power
**Contrast: NMES+AO versus NMES+LO**						
S2-D3	3.516	0.714	4.926	0.000	0.000	0.379
S4-D3	3.903	0.909	4.295	0.000	0.000	0.298
S4-D5	4.036	0.880	4.584	0.000	0.000	0.307
**Contrast: NMES+AE versus NMES+LO**						
S1-D1	2.212	0.856	2.585	0.010	0.033	0.316
S1-D2	2.515	0.707	3.556	0.000	0.002	0.382
S2-D2	3.249	0.802	4.052	0.000	0.000	0.337
S2-D3	3.756	0.706	5.318	0.000	0.000	0.383
S3-D3	2.397	0.882	2.717	0.007	0.024	0.306
S4-D3	3.789	0.920	4.119	0.000	0.000	0.294
S4-D5	3.655	0.884	4.135	0.000	0.000	0.306
S4-D6	2.819	0.986	2.858	0.005	0.017	0.274
S6-D7	2.743	1.080	2.541	0.012	0.036	0.250
**Contrast: NMES+AI versus NMES+LO**						
S1-D1	3.509	0.864	4.062	0.000	0.000	0.313
S1-D2	4.013	0.714	5.618	0.000	0.000	0.379
S2-D2	4.653	0.839	5.546	0.000	0.000	0.322
S2-D3	4.728	0.718	6.587	0.000	0.000	0.377
S4-D3	4.870	0.923	5.277	0.000	0.000	0.293
S4-D5	4.138	0.896	4.617	0.000	0.000	0.302
S4-D6	2.958	0.975	3.034	0.003	0.011	0.277
S6-D5	3.694	1.187	3.113	0.002	0.009	0.228
S6-D8	2.983	1.134	2.630	0.009	0.030	0.238

FDR, false discovery rate; HbO, oxygenated hemoglobin; NMES, neuromuscular electrical stimulation; AO, action observation; AE, action execution; AI, action imitation; LO, landscape observation. Only channels meeting false-discovery corrections of *P*_FDR_ < 0.05 are shown.

### 3.2. Regions of interest with significant activation compared with the control condition

**Figure 4:**
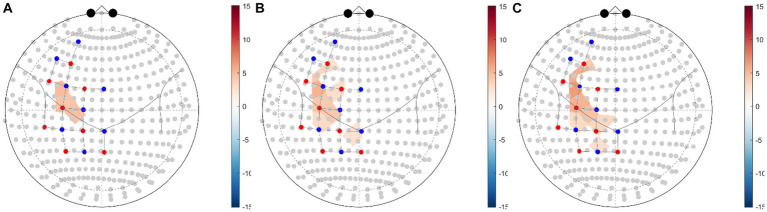
fNIRS ROI brain activity maps based on HbO data for **(A)** NMES+AO versus NMES+LO, **(B)** NMES+AE versus NMES+LO, **(C)** NMES+AI versus NMES+LO. The color of the ROI of the T-statistic according to the color bar shows ROIs significant at a false-discovery rate of *P*_FDR_ < 0.05 corrected for all comparisons (31, 53). ROI, region of interest; FDR, false discovery rate; HbO, oxygenated hemoglobin; NMES, neuromuscular electrical stimulation; AO, action observation; AE, action execution; AI, action imitation; LO, landscape observation.

The GLM regression coefficients (β) of HbO data in each condition across all channels were averaged by ROI weights (Table 2). The regression coefficients of each brain region in each experimental condition compared with the control condition were calculated (Table 4; Figure 4). The group-level GLM analysis showed that 2 ROIs were significantly activated (*P*_FDR_ < 0.05) in the NMES+AO condition, including BA6 and BA44 (Figure 4A); 5 ROIs were significantly activated (*P*_FDR_ < 0.05) in the NMES+AE condition, including BA6, BA40, BA44, BA45, and BA46 (Figure 4B); 6 ROIs were significantly activated (*P*_FDR_ < 0.05) in the NMES+AI condition, including BA6, BA7, BA40, BA44, BA45, and BA46 (Figure 4C). For most ROIs, the activation amplitude from high to low in turn was NMES+AI, NMES+AE, and NMES+AO (Table 4; Figure 4).

**Table 4:** ROIs with significant increases (*P*_FDR_ < 0.05) in HbO concentration compared with the control condition.

ROI	Beta (ꭒM)	SE	*t*-value	*p*-value	*P*_FDR_	Power
**Contrast: NMES+AO versus NMES+LO**						
BA44	1.594	0.603	2.642	0.009	0.014	0.161
BA6	2.532	0.495	5.112	0.000	0.000	0.000
**Contrast: NMES+AE versus NMES+LO**						
BA40	2.184	0.659	3.315	0.001	0.002	0.049
BA44	3.227	0.586	5.507	0.000	0.000	0.000
BA45	2.848	0.583	4.888	0.000	0.000	0.001
BA46	2.400	0.635	3.777	0.000	0.000	0.017
BA6	2.678	0.493	5.428	0.000	0.000	0.000
**Contrast: NMES+AI versus NMES+LO**						
BA40	2.437	0.656	3.712	0.000	0.001	0.020
BA44	4.244	0.601	7.057	0.000	0.000	0.000
BA45	4.211	0.591	7.121	0.000	0.000	0.000
BA46	3.514	0.645	5.450	0.000	0.000	0.000
BA6	3.565	0.499	7.146	0.000	0.000	0.000
BA7	2.036	0.711	2.864	0.005	0.007	0.113

ROI, region of interest; FDR, false discovery rate; HbO, oxygenated hemoglobin; BA, Brodmann Area; NMES, neuromuscular electrical stimulation; AO, action observation; AE, action execution; AI, action imitation; LO, landscape observation. ROI analysis shows significant activations at a false-discovery rate of *q* < 0.05. The left Brodmann areas BA6, BA7, BA40, BA44, BA45, and BA46 were examined as the six regions in the analysis. Only regions showing statistical changes are reported (31).

## 4. Discussion

### 4.1. Activation of brain areas during neuromuscular electrical stimulation synchronized mirror neuron rehabilitation strategies

In this study, fNIRS was used to explore the cortical activation patterns induced by NMES combined with three common MNS rehabilitation strategies (AO, AE, AI). The results showed that NMES+AO, NMES+AE, and NMES+AI all activated the brain areas, including the MNS, suggesting that the simultaneous application of NMES and AO, AE, or AI can improve the treatment effects by inducing cortex activation and neuroplasticity. According to the MNS theory, when participants observe the movements, the activation of MNS may induce automatic “imitation” in the brain unconsciously, resulting in a certain degree of pre-activation of the corresponding neural pathway controlling the movement of the target muscles, thereby exhibiting a positive impact on motor learning or function (35). In addition to brain activation, NMES and FES could also evoke muscle contraction, joint movement, and sensory input to the central nervous system, thus establishing a feedback loop (59, 60). A previous study showed that the combination of peripheral ES (PES) and AO was helpful for improving neural plasticity and treatment effects (61). Thus, there is a possibility to improve brain activation and clinical outcomes by combing NMES with MNS rehabilitation strategies. On the other hand, compared with pure NMES treatment, the new therapeutic strategies of NMES+AO, NMES+AE, and NMES+AI could give patients a better treatment experience, fostering increased interest and enthusiasm to achieve better treatment effects. Therefore, these innovative rehabilitation treatment approaches hold great potential and are deserving of promotion in clinical practice.

Our data showed that compared to the NMES+AO condition, more brain areas were involved in the NMES+AE and NMES+AI conditions. For most ROIs, the elicited activation amplitudes of MNS in decreasing order were NMES+AI, NMES+AE, and NMES+AO, suggesting that active therapeutic exercise may lead to better curative effects than passive exercise (such as passive movement induced by NMES) or passive observation in neurorehabilitation. Existing literature has shown that the activation of MNS induced by AI and AE is stronger than that of AO when observing a tester perform a clean-up task (19). A recent study showed that AE-evoked brain activation was stronger than that of MI (62). Except for amplitude difference, it has also been reported that MNS exhibits a certain of lateralization, and there are differences between AO and AE in lateralization. Specifically, AO generally triggers the activation of bilateral hemispheres, while AE has a higher activation level in the contralateral hemisphere (63). A recent study showed that sensorimotor activation patterns in AO, MI, and AO + MI differ between individuals, and that EEG features can be used to guide the intervention protocol for enhancing the cortical activation (64).

This study is novel in that it provides some new alternative rehabilitation interventions based on the combination of MNS and NMES, which simultaneously combines central and peripheral stimulation. The fNIRS brain imaging technology was used to quantitatively and accurately measure brain activity related to these new methods of rehabilitative treatments in real clinical settings. In our hypothesis, observing, executing, and imitating related actions during NMES might induce “central-peripheral synchronous stimulation” to enhance the activation of MNS and the recruitment of MU, thereby improving the recovery of neuromuscular control function. A previous pain study revealed that compared with top-down brain modulation, bottom-up ES could activate similar brain areas, and the proprioceptive signals activate the somatosensory cortex and the motor function-relevant neuronal network (65). The NMES had been shown to induce plastic changes in patients with stroke. A recent fNIRS study in stroke patients with aphasia revealed that speech therapy combined with NMES on median nerve could enhance cortical activation and functional connectivity to achieve higher clinical efficacy (66). In addition, NMES also affects the sensorimotor network-related brain regions, which may be helpful for the activation of MNS. A recent fMRI study in healthy participants revealed that sensory observation had a similar activation pattern as somatosensory stimulation, which might be beneficial to sensorimotor dysfunction recovery (67). These findings indicate that the combination of NMES and MNS-based rehabilitation methods could not only enhance the activation of MNS and related brain regions at the brain level but also improve neuromuscular control and MU recruitment at the peripheral level. Simultaneously stimulus from the peripheral and central directions might boost each other to maximize the activation of relevant brain regions, promote neuroplasticity and achieve a synergistic therapeutic effect.

### 4.2. Potential application of BCI based on MNS theory and fNIRS signals in rehabilitation medicine

In recent years, BCI related to MNS and ES rehabilitation systems have made great progress. BCI is the interface between the brain and a machine or computer, which can bypass peripheral nerves to transmit signals between the nervous system and external devices (68). BCI can be used in the rehabilitation field by obtaining, decoding, and modeling brain activation patterns and using them to interact with computers and rehabilitation devices (61, 69). There are many advantages in using fNIRS-based hemodynamic responses to control a BCI rehabilitation system, especially in the AE task type and lower limb movements (70, 71).

In this study, the obvious activation patterns were observed with fNIRS, providing a possible implementation method of BCI based on fNIRS and neurofeedback therapy. Since most patients who need the BCI rehabilitation system have usually lost the ability to perform active exercise, physiological signals generated by brain activity in the process of AO and MI are increasingly being used as effective control signals, and these signals are likely to be generated by MNS excitement. For example, mu (μ) suppression is an important electrophysiological evidence for the existence of MN, and a large number of BCIs use this EEG signal as a control signal (72). The haptic feedback induced by ES can also evoke SMC brain activations and can be used as control signals in the BCI system (43). A previous study showed that AO-FES integrated BCI can more strongly activate brain regions than AO (3). When designing an MI-based BCI, it should to noted that AE produces a higher activation amplitude at a faster speed than MI (62). A recent study showed that feedback provided by a BCI-AO system combined with PES was helpful to enhance brain activation of SMC in patients with stroke (61). In addition, BCI-based FES might help patients with low BCI performance to participate in BCI-based rehabilitation treatment, thus improving the treatment effects (69). Recently, new ideas about rehabilitation BCI have emerged, and non-invasive brain-spine interface is a promising direction among them (73). Based on the above experimental results and related literatures, fNIRS has the potential to be used as control signals for the design, development, and application of BCI rehabilitation systems for both upper limb and lower limb motor functions.

In the future, fNIRS-BCI rehabilitation equipment can be developed based on relevant data and experience (74). Compared with fMRI and other brain imaging technologies, fNIRS is characterized by high ecology, anti-motion artifact, real-time imaging, low cost, and ease to combine with other technologies for multimodal functional imaging. Real-time optical morphologies of cortical hemodynamic responses can be obtained from multiple measurement locations simultaneously (40). The above technical characteristics make fNIRS suitable for the evaluation of MNS activation, and fNIRS may be the best technique to evaluate the mechanism of AE brain activation (49). The application of AOT in lower limb motor function, gait, and ADL has been gradually gaining attention, and fNIRS can be used to detect the brain activation patterns in these conditions (75–77). For example, fMRI was used to detect the activation patterns of MNS during gait observation (78). However, the fMRI experiment of lower limb movement can only be used for observation tasks. The fNIRS technique is widely used in brain activation related to gait and posture control and is helpful for promoting brain mechanism research of lower limb AOT and other lower limb-related MNS rehabilitation strategies (79, 80).

### 4.3. Future perspectives and study limitations

In this study, to improve the standardization and homogenization of the experiment, the time of occurrence of the stimulus was precisely controlled by a computer program, which can provide a reference for the formulation of experimental paradigms in the future. The determination of the MNS experimental paradigm is very important for the standardization of rehabilitation evaluation, which has not been unified yet. In AOT and AO-based BCI, the commonly used observation methods include video recording, mirror visual feedback, a demonstration by others or therapists, movement of a healthy body, and animation. Evaluation and treatment can be performed with the help of a TV, computer, tablet computer, mirror, and self-developed equipment (81, 82). In the future, it is very necessary to establish standardized and computerized experimental paradigms to promote the homogeneity and reusability of various research results.

At present, the activation mode of brain regions induced by upper limb movement related to MNS and ES has been studied extensively. Recently, AOT studies of lower limb and oral movements, such as gait and swallowing movement observation, have gained increasing attention, but the brain activation patterns of these techniques combined with ES still needs further exploration (23, 78). Another important research direction is the choice of new analytical methods. With the development of data analysis technologies and methods, some new analysis methods should be used to get a deeper understanding of the neural mechanism of MNS and NMES; for example, resting-state functional connectivity (FC), task-based dynamic FC, network control theory, executive control network, and new algorithms based on machine learning may be the future directions of this study (83, 84).

There are also some limitations and shortcomings to be considered in this study. First, the number of optodes of the fNIRS device used in this study is limited, and only a few brain regions could be covered and detected, mainly the left MNS. In future studies, we can use fNIRS systems with more probes to cover more brain regions, which allows for more ROIs and even the whole brain to be detected. This can also lead to the observation of bilateral changes and lateralization, as well as the interaction between MNS and related brain regions and brain functional networks (85). Second, due to limitations in its technical characteristics, the fNIRS technique can only explore cortical activation and thus, the detection of subcortical activation is impossible. In addition, unlike fMRI, fNIRS cannot obtain structural images, though multimodal functional imaging is suitable for understanding more detailed mechanisms (86). Third, most of the participants are healthy young volunteers, and there might be some differences in brain activation in populations with different age groups and health conditions (3, 56, 87). In addition, to further prove the feasibility of applying the results of this study to patients with central nervous system injuries, the same experiment should be conducted in a relevant patient population with age and gender-matched healthy controls to explore the different cerebral activation patterns between patients and healthy participants. In addition, a clinical trial should be conducted to test the effects of these new rehabilitative treatment strategies.

In summary, the MN theory has become an important neuroscience basis for numerous rehabilitation strategies and treatment techniques. We demonstrated that the MNS can be activated during NMES synchronized with AO, AE, and AI, which indicates that the synchronous application of NMES and mirror neuron rehabilitation strategies might be feasible in clinical rehabilitation. In addition, fNIRS signal patterns of the brain regions including MNS induced by NMES+AO, NMES+AE, and NMES+AI might be helpful for the design, development, and clinical application of MNS and fNIRS-based BCI and neurofeedback therapy. The brain activation area and amplitude can be improved by combining the bottom-up NMES with top-down active rehabilitation strategies based on MNS theory.

## Data availability statement

The raw data supporting the conclusions of this article will be made available by the authors, without undue reservation.

## Ethics statement

The requirement of ethical approval was waived by Medical Ethics Committee of the China Rehabilitation Research Center for the studies involving humans because this study was approved by the Medical Ethics Committee of the China Rehabilitation Research Center (approval number: 2021-053-1). The studies were conducted in accordance with the local legislation and institutional requirements. Written informed consent for participation was not required from the participants or the participants’ legal guardians/next of kin because all participants signed the informed consent form according to the Helsinki Declaration. Written informed consent was obtained from the individual(s) for the publication of any potentially identifiable images or data included in this article.

## Author contributions

YC and FC contributed to the conception and design of the study. FH guided the experimental design. YC and RY contributed to the recruitment of participants. YC programmed the experiment program, carried out the experiment, and did the data acquisition, performed data analysis and interpretation and wrote the first draft of the manuscript. All authors contributed to the article and approved the submitted version.

## Funding

This study was supported by Capital’s Funds for Health Improvement and Research Youth Fund (no. CFH2022-4-6014) and China Rehabilitation Research Center Youth Fund (no. 2021ZX-Q8).

## Conflict of interest

The authors declare that the research was conducted in the absence of any commercial or financial relationships that could be construed as a potential conflict of interest.

## Publisher’s note

All claims expressed in this article are solely those of the authors and do not necessarily represent those of their affiliated organizations, or those of the publisher, the editors and the reviewers. Any product that may be evaluated in this article, or claim that may be made by its manufacturer, is not guaranteed or endorsed by the publisher.
