# Supplementary material for: Cortical activation of neuromuscular electrical stimulation synchronized mirror neuron rehabilitation strategies: an fNIRS study
Source: Front Neurol. 2023 Aug 4;14:1232436. doi: 10.3389/fneur.2023.1232436 (PMC10437114; doi:10.3389/fneur.2023.1232436)
Supplement: Supplementary file 1 [file Data_Sheet_1.docx]

Supplementary Material

Cortical activation of neuromuscular electrical stimulation synchronized mirror neuron rehabilitation strategies: an fNIRS study

Yao Cui^1,2*^, Fang Cong^1,2*^, Fubiao Huang^2,3^, Ming Zeng^4^, Ruxiu Yan^2,3^

*** Correspondence:** Fang Cong: [congfang@crrc.com.cn](mailto:congfang@crrc.com.cn); Yao Cui [cuiyao@crrc.com.cn](mailto:cuiyao@crrc.com.cn)

## Supplementary Figure


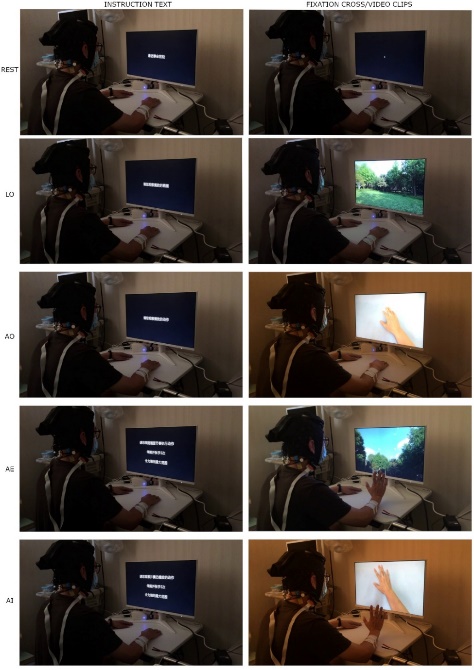


**Supplementary Figure 1.** Experiment Setup. The participant wearing a fNIRS cap sat in a chair in front of a display screen placed on a height adjustable table with two NMES electrodes attached on his right extensor digitorum communis. The instruction text and contents to display on the screen differs in different experiment conditions. During rest phase, a fixation cross was displayed on the center of the screen; during LO and AE phases, landscape videos were displayed on the full screen; during AO and AI phases, action videos were displayed on the full screen. The participant agreed to use his photos in the manuscript for non-commercial use and signed a consent form to authorize the use of his portrait right.

Abbreviations: LO, landscape observation; AO, action observation; AE, action execution; AI, action imitation.
